# Semen quality of 4480 young cancer and systemic disease patients: baseline data and clinical considerations

**DOI:** 10.1186/s12610-016-0031-x

**Published:** 2016-02-18

**Authors:** Jacques Auger, Nathalie Sermondade, Florence Eustache

**Affiliations:** Service d’Histologie-Embryologie, Biologie de la Reproduction/CECOS, Hôpitaux Universitaires Paris Centre, Site Port-Royal, 53, Avenue de l’Observatoire, 75014 Paris, France; INSERM U1016, Equipe “Génomique, Epigénétique et Physiologie de la Reproduction”, Institut Cochin, Université Paris Descartes, Paris, France; Service d’Histologie-Embryologie, Cytogénétique, Biologie de la Reproduction/CECOS, Hôpitaux Universitaires Paris Seine-Saint-Denis, Site Jean Verdier, 93143 Bondy, France

**Keywords:** Cancer, Systemic disease, Semen quality, Sperm cryopreservation, Sperm banking, Assisted reproductive technologies, Cancer, Maladie auto-immune, Qualité du sperme, Conservation du sperme, Congélation, Aide médicale à la procréation

## Abstract

**Background:**

Except for testicular cancer and Hodgkin’s disease, baseline data on semen quality in case of cancers as well as systemic pathologies of the young adult are scarce or based on low sample size.

**Methods:**

Semen quality in patients having testicular cancer (TGCT, *n* = 2315), Hodgkin’s disease (HD, *n* = 1175), non-Hodgkin’s lymphoma (NHL, *n* = 439), leukemia (L, *n* = 360), sarcoma (S, *n* = 208), brain tumour (BT, *n* = 40), Behcet’s disease (Behcet’s, *n* = 68) or multiple sclerosis (MS, *n* = 73) was studied and compared to that of 1448 fertile men candidates for sperm donation (CSD) and 208 partners of pregnant women (PPW). All samples were studied following the same methodology in a single laboratory. Post freezing and thawing semen characteristics were also studied.

**Results:**

The percentage of normozoospermic men was only 37 % for L patients and lower than 60 % for TGCT, NHL, S and BT. The level of sperm production was differently decreased according to pathologies, the median total sperm count in TC and L patients being four times lower (*p* < 0.01 when compared to CSD and PPW). The lowest percentage of progressively motile spermatozoa was found for L and BT patients (both, *p* < 0.01 compared to CSD and PPW). The percentage of morphologically normal spermatozoa was also reduced in cancer patients, especially in BT patients. Progressive motility after thawing in patients was about half that observed among candidates for sperm donation. In almost half of the semen of patients with testicular cancer or leukemia, the total number of motile spermatozoa per straw was less than 0.5 × 10^6^ compared to 4.3 × 10^6^ in CSD.

**Conclusions:**

The present data confirm on large series the deleterious impact of various cancers of the young adult on semen quality, establishing thus baseline data for future studies. Owing to the post-thaw quality of the frozen straws, future fertility projects for the majority of the patients studied (in case there is no post-treatment recovery of spermatogenesis) should necessitate an ICSI to provide the best chance of paternity whatever the fertility check-up in the female partner.

## French language Abstract

**Contexte:**

En dehors du cancer du testicule et de la maladie de Hodgkin, les données de la littérature sur la qualité du sperme dans le cas de cancers et de maladies systémiques du jeune adulte sont rares et le plus souvent basées sur de faibles effectifs.

**Méthodes:**

La qualité spermatique de patients ayant un cancer du testicule (TGCT, *n* = 2 315), une maladie de Hodgkin (HD, *n* = 1175), un lymphome non Hodgkinien (LNH, *n* = 439), une leucémie (L, *n* = 360), un sarcome (S, *n* = 208), une tumeur cérébrale (BT, *n* = 40), une maladie de Behcet (Behcet, *n* = 68) ou une sclérose en plaque (MS, *n* = 73) a été étudiée et comparée à celle de 1448 hommes féconds candidats au don de spermatozoïdes (CSD) et 208 partenaires de femmes enceintes (PPW), utilisant la même méthodologie dans un seul laboratoire. Les caractéristiques du sperme après dégel ont également été analysées.

**Résultats:**

Le pourcentage de spermes normozoospermiques était seulement de 37 % chez les patients L, et < 60 % pour les patients TGCT, NHL, S et BT. La production spermatique était diminuée dans la plupart des pathologies, le nombre total spermatozoïdes par éjaculat des patients TC et L étant 4 fois plus faible (*p* <0,01 par rapport aux hommes féconds). Le plus faible pourcentage de spermatozoïdes mobiles a été trouvé pour les patients L et BT (*p* <0,01 par rapport aux hommes féconds). Une diminution du pourcentage de spermatozoïdes normaux a aussi été observée chez les patients cancéreux, particulièrement pour les patients BT. La motilité progressive après décongélation des patients était diminuée de moitié par rapport aux candidats pour le don de sperme. La médiane du nombre total de spermatozoïdes mobiles par paillette était inférieure à 0,5 × 10^6^ pour TGCT et L contre 4,3 × 10^6^ pour les candidats au don.

**Conclusion:**

Les données présentées obtenues sur de grandes séries rarement publiées constituent des données de référence pour de futures études. Dans le cas d’une utilisation de paillettes en AMP, l’ICSI sera nécessaire dans la majorité des cas.

## Background

Advancements in early diagnoses and new treatments of cancer have greatly contributed to a high survival rate in the last decades [1]. With the increasing number of young adults survivors of cancer, long-term quality of life has become an important issue, especially preservation of reproductive potential [2]. The same issues are observed for men presenting severe immunological diseases, such as multiple sclerosis. Available literature about semen quality of men concerned with those chronic tumoral or systemic diseases is usually scarce, often based on low sample sizes of patients studied. Studies based on high number of patients have been recently reported [3, 4], especially about semen quality of testicular cancer and Hodgkin’s disease patients, the most frequent cancers in young men [5, 6]. The aggregation of different pathologies in heterogeneous groups (such as, ‘hematological cancer’, ‘other cancers’ etc.) is one limitation of several studies in the field because these pathologies have different origins, may have different impacts on individuals and, maybe, a different effect on the testis and the male reproductive tract. Another frequent limitation of previous reports is the absence of reference population in order to appreciate the magnitude of the alterations in semen quality related to the diseases.

Testicular and post-testicular disorders are well-known side effects of anti-tumoral treatments and other therapies used for immunological diseases [7]. These alterations may be transient, but also long lasting [8, 9]. The middle term and long term impact of these treatments is unpredictable in most cases, thus, sperm cryopreservation remains the cornerstone of male fertility preservation in patients treated for these pathologies [10, 11]. However, little has been reported on the practical usefulness of sperm banking according to the various indications.

The objectives of the present study, based on a 30-year period in sperm banking for cancer and systemic disease patients, are (i) to compare the sperm characteristics in patients and healthy fertile men, and, (ii) to assess the feasibility of using the cryopreserved semen samples in the various pathologies studied according to currently available Assisted Reproductive Technologies.

## Methods

The present study is a retrospective observational study.

### Men under study

Two categories of men were studied : (i) patients with various types of cancer or auto-immune diseases who were referred for sperm cryopreservation before a potentially gonadotoxic treatment to the former Bicêtre University Hospital sperm bank (Centre d’Etude et de Conservation des Œufs et du Sperme humains, CECOS) transferred to the Cochin university hospital in 1994 (study period: 1974–2003), and, (ii) two populations of healthy fertile men recruited in the same sperm bank during the same time period, corresponding to a population of 1448 candidates for sperm donation (CSD) (<45 years old and being fathers, as required by French law) and another group of 208 healthy men partners of pregnant women (PPW) volunteers to participate in a study evaluating the impact of environmental factors on semen quality and time to pregnancy.

Pathologies requiring potentially gonadotoxic treatments, and therefore sperm banking, were testicular germ cell tumour cancer (TGCT, *n* = 2315) including seminoma and non-seminoma tumours, the vast majority of patients being referred before orchidectomy, Hodgkin’s disease (HD, *n* = 1175), non-Hodgkin’s lymphoma (NHL, *n* = 439), leukemia (L, *n* = 360) including both acute and chronic forms, sarcoma (S, *n* = 208) including bone and soft tissue sarcoma, brain tumour (BT, *n* = 40) of various histological nature, and, two systemic diseases, Behcet’s disease (Behcet’s, *n* = 68) and multiple sclerosis (MS, *n* = 73). Patients with a history of previous gonadotoxic treatment (<5 %) were excluded. Age at referring, and fertility history were recorded.

### Semen analysis

All semen samples were collected by masturbation in the laboratory. A period of sexual abstinence of 3–5 days before semen collection was recommended to all patients and healthy men, and the accurate period of sexual abstinence in days was recorded at the time of semen collection. Standardized procedures for routine semen analysis were used throughout the study period. Briefly, semen samples were incubated at 37 °C and analysed within one hour. Seminal volume was determined by weighing. Sperm concentration (x10^6^ per ml) was assessed using a haemocytometric method. The total number of spermatozoa per ejaculate (x10^6^), grossly reflecting testicular sperm production, was calculated as the product of sperm concentration by the volume of seminal fluid. The percentage of progressively motile spermatozoa was assessed at 37 °C at x100 and x400 magnification with phase optics in four to six fields chosen at random, in two preparations, the mean value being reported. The percentage of morphologically normal spermatozoa was assessed according to the classification of David [12], slightly modified in the late 1990s [13], the criteria used for scoring the normal spermatozoa remaining the same considering borderline aspects as normal, an equivalent to the ‘liberal’ WHO criteria [14].

### Sperm cryopreservation

Except for partners of pregnant women, all semen samples were frozen according to a standardized slow-freezing method as previously reported (see for example [15]). Briefly, each semen sample was diluted into a cryoprotectant medium and this preparation was distributed into French straws. Freezing was carried out in liquid nitrogen vapours using an automatic freezer with a programmed cooling rate. The straws were directly plunged into liquid nitrogen at the end of the freezing process. In order to evaluate the tolerance to freezing, one straw per sample was thawed to assess the post-thaw progressive motility in order to calculate the recovery rate in progressive motile spermatozoa (corresponding to the ratio of post thaw motility to pre freeze motility, expressed in %) and the total number of progressive motile spermatozoa per straw (NMSPS, accounting for straw volume, dilution ratio, sperm concentration and post thaw motility, ×10^6^). Of note, both the type of straw and the cryoprotectant have been changed during the 1990s: 0.30 ml high security French straw has replaced the classical 0.25 ml French straw and, a HEPES buffered - ready-to-use - freezing medium has replaced the glycerol-egg yolk-citrate medium used before. However, before these changes it was verified that the sperm characteristics after freezing and thawing were not significantly different with the new material and medium in comparison to the classical ones.

### Statistical analysis

In this study, only the first semen sample collected either in patients or fertile men was considered for descriptive and comparative statistical analyses. All statistics were performed with the BMDP statistical software (Statistical Solutions, Cork, Ireland). Quantitative values were expressed as mean ± standard deviation and/or median with interquartile (IQ) ranges. The percentage of normozoospermic men according to WHO reference values (≥39 × 10^6^ per ejaculate and ≥32 % of progressive motility [16], excluding morphology) was calculated in order to appreciate the extent of men with an acceptable fertility potential for each pathology studied in comparison to fertile men. In addition, the similarity of the mean values for various semen characteristics between all groups of patients and healthy men studied was assessed by a one-way analysis of variance (BMDP 7D subroutine), taking unequal variances into account (Brown-Forsythe test) when necessary. When the null hypothesis was rejected, post-hoc Tukey tests were used for pair-wise comparisons between patients and healthy fertile men.

Semen collection failure and azoospermia rates were recorded in order to assess the feasibility of sperm freezing in the various groups of pathologies. To discuss the potential use of the frozen sperm samples through Assisted Reproductive Technologies (ART), patients were stratified according to their pathology and to NMSPS categories, defined by a possibly minimal NMSPS required for each strategy: ≥ 4x10^6^ for Intra-Uterine Insemination (IUI) [17], in the range < 4 × 10^6^ and ≥2 × 10^6^ for conventional In Vitro Fertilization (IVF) and < 2 × 10^6^ for Intra Cytoplasmic Sperm Injection (ICSI).

### Ethical approval

The study was approved by the Cochin University Hospital for the three groups of men studied (CSD, PPW and cancer patients). Informed consent was obtained from all participants.

## Results

Mean age of the patients was 28 ± 7 years old, 12.9 % of the patients being 20 years old or younger. Thirty seven percent of all patients (*n* = 1645) were childless. Table 1 summarizes the age and percentage of childless patients for the various pathological conditions studied.

**Table 1:** Age and fertility status of patients according to the various pathological conditions studied

	TGCT	HD	NHL	L	S	BT	Behcet	MS
*n*	2315	1175	439	360	208	40	68	73
Age (year, median)	28.8	26	28.8	27.5	23.1	28.2	29.7	30.3
% ≤ 20 year-old	7.1	19.1	15.7	20.6	35.6	25	5.9	5.5
% Childless	53	61.3	54.1	54.1	75.4	72.5	39.7	24.7

*TGCT* testicular germ cell tumour, *HD* Hodgkin’s disease, *NHL* non-Hodgkin’s lymphoma, *L* leukemia, *S* sarcoma, *BT* brain tumour, *Behcet* Behcet’s disease, *MS* multiple sclerosis

### Semen quality

**Fig. 1:**
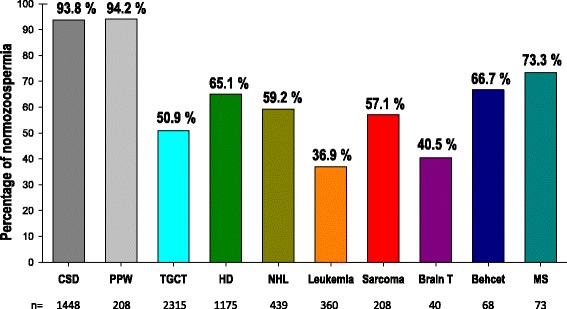
Percentage of normozoospermic men in the various pathological conditions and the two groups of healthy fertile men (CSD and PPW)

Median sexual abstinence was in the range of a minimum of 2 days and a maximum of 7 days [18] while about one quarter of the patients in all the pathologies studied had a longer period of sexual abstinence than one week (data not shown). Figure 1 presents the percentage of normozoospermic patients in the various pathological groups and healthy men according to WHO 2010 reference values [16]. Normozoospermia was observed for only half of TGCT patients, and 40 % or lower for L and BT patients, compared to more than 93 % in both groups of healthy fertile men.

**Fig. 2:**
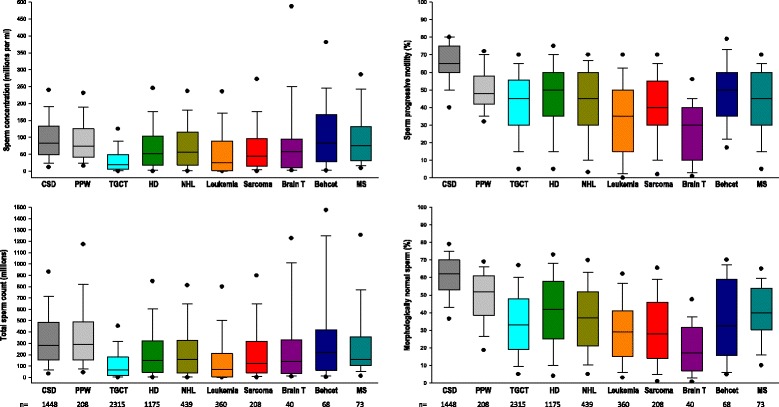
Box plot displaying the 10th, 25th, 50th, 75th, and 90th percentile values and the extreme 5th and 95th percentiles (*circles*) of sperm concentration, total sperm count, progressive sperm motility and normal sperm morphology according to the various pathological conditions. Distributions in healthy fertile men (CSD and PPW) are presented for comparison (see Table 2 for statistical comparisons)

A statistically significant lower sperm concentration, total sperm count, progressive motility and normal morphology was observed for most of the pathological groups of men studied in comparison to both groups of healthy fertile men (Fig. 2 and Table 2). Overall, TGCT and leukemia patients had the lowest semen characteristics and patients with a systemic disease had the best semen quality among all pathologies studied. Notably, the level of sperm production in TGCT and leukemia patients was about one quarter of the level found in CSD and PPW.

**Table 2:** Semen characteristics in the various pathological groups of men studied and comparisons with the two groups of healthy fertile men

	CSD	PPW	TGCT	HD	NHL	L	S	BT	Behcet	MS	F	*p*-value
*n*	1448	208	2315	1175	439	360	208	40	68	73	-	-
Seminal volume (ml)	3.8 ± 1.8^a^	4.2 ± 2.0	3.7 ± 2.0 ^***^	3.2 ± 1.9^**,****^	3.1 ± 1.8^**,****^	2.9 ± 1.7^**,****^	3.2 ± 1.8^**,****^	3.5 ± 1.8	3.2 ± 1.9 ^****^	3.2 ± 1.9^*,****^	19	<0.0001
	3.5 (2.5–4.8)^b^	3.9 (2.7–5.2)	3.4 (2.3–4.8)	2.9 (1.9–4.1)	2.9 (1.9–4.1)	2.6 (1.7–3.9)	2.9 (2.0–4.3)	3.1 (2.1–4.4)	2.8 (1.5–4.6)	2.9 (1.7–4.5)		
Sperm concentration (×10^6^/ml)	99.1 ± 73.8	94.2 ± 71.8	36.1 ± 48.5^**,****^	79.4 ± 103.1^**^	81.2 ± 91.7^**^	63.0 ± 96.3^**,****^	72.8 ± 83.2^**^	86.7 ± 118.4	116.6 ± 125.8	98.3 ± 87.4	118	<0.0001
	82.4 (48.4–133.0)	74.0 (40.6–125.0)	19.6 (5.4–48.8)	52.0 (18.1–104.0)	55.6 (18.0–115.2)	25.6 (1.6–88.0)	44.7 (15.9–96.5)	56.8 (9.8–94.6)	83.2 (27.6–167.7)	74.6 (30.6–131.7)		
Total sperm count (×10^6^)	362 ± 328	383 ± 350	127 ± 174^**,****^	250 ± 388^**,****^	245 ± 289^**,****^	179 ± 293^**,****^	233 ± 307^**,****^	287 ± 394	357 ± 428	312 ± 439	89	<0.0001
	281 (152–486)	291 (153–488)	64 (16–178)	147 (45–323)	160 (38–324)	71 (4–209)	124 (39–316)	140 (34–331)	221 (63–419)	159 (105–357)		
Progressively motile sperm (%)	65 ± 12^****^	50 ± 13^**^	43 ± 19^**,****^	47 ± 20^**^	43 ± 21^**,****^	33 ± 21^**,****^	41 ± 19^**,****^	27 ± 17^**,****^	47 ± 18^**^	43 ± 19^**,***^	300	<0.0001
	65 (60–75)	48 (42–58)	45 (30–56)	50 (35–60)	45 (30–60)	35 (15–50)	40 (30–55)	30 (10–40)	50 (35–60)	45 (30–60)		
Morphologically normal sperm (%)	61 ± 13 ^****^	49 ± 15^**^	34 ± 19^**,****^	41 ± 21^**,****^	37 ± 20^**,****^	29 ± 18^**,****^	31 ± 20^**,****^	20 ± 15^**,****^	36 ± 22^**,****^	41 ± 16^**,****^	313	<0.0001
	62 (53–70)	52 (39–61)	33 (19–48)	42 (25–58)	37 (21–52)	29 (15–41)	28 (14–46)	17 (7–32)	33 (16–59)	40 (30–54)		

For all pathological conditions, differences between groups of men were investigated by one-way analysis of variance. For pair-wise comparisons, post hoc Tukey tests were carried out, with: * *p* < 0.05 and ** *p* < 0.01 in comparison to CSD and ***p < 0.05 and ****p <0.01 in comparison to PPW

^a^Mean ± SD; ^b^Median (IQ range)

### Clinical considerations

**Fig. 3:**
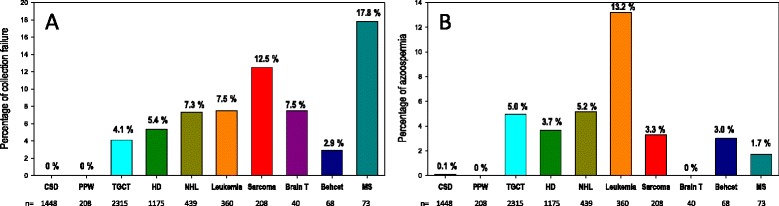
Percentage of patients with semen collection failure (**a**) and azoospermia (**b**)

What was the proportion of men referred for sperm cryopreservation who could not effectively benefit from this procedure? This essentially concerned men who could not succeed in collecting a semen sample and men with azoospermia at the time of their referral. Sample collection failure concerned 5.7 % of men, varying from 2.9 % in Behcet’s disease to 17.8 % in multiple sclerosis. Overall, azoospermia was diagnosed for 5.7 % of men at the time of their referral, the highest rate being for leukemia patients (13.2 %) (Fig. 3).

Do the studied pathologies affect tolerance to freezing and thawing in comparison to healthy men (CSD)? The post thaw progressive motility, the motility recovery rate and the number of motile sperm per straw were significantly lower for the vast majority of patients and particularly for TGCT and L patients (Table 3).

**Table 3:** Post thaw sample characteristics according to the various pathologies studied in reference to the CSD healthy group

	CSD	TGCT	HD	NHL	L	S	BT	Behcet	MS	F	*p*-value
*n*	1448	2315	1175	439	360	208	40	68	73	_	_
Post-thaw motility (%)	40 ± 14	21 ± 15^*^	24 ± 16^*^	23 ± 16^*^	17 ± 15^*^	22 ± 15^*^	17 ± 14^*^	26 ± 15^*^	23 ± 17^*^	223	<0.0001
Motility recovery rate (%)	60 ± 17	44 ± 25^*^	46 ± 25^*^	46 ± 25^*^	40 ± 28^*^	47 ± 26^*^	53 ± 29	52 ± 21	51 ± 27^*^	73	<0.0001
NMSPS (×10^6^)	4.75 ± 3.21	1.26 ± 1.80^*^	2.78 ± 3.44^*^	2.54 ± 2.86^*^	1.80 ± 3.12^*^	2.06 ± 2.76^*^	4.32 ± 6.96	4.59 ± 5.90	2.75 ± 2.62^*^	156	<0.0001
Number of straws (median)	_	21	19	20	15	18	15	20	23	_	_

For all pathological conditions, differences between groups of men were investigated by one-way analysis of variance. For pair-wise comparisons, post hoc Tukey tests were carried out, with: ^*^ *p* < 0.01 in comparison to CSD

**Fig. 4:**
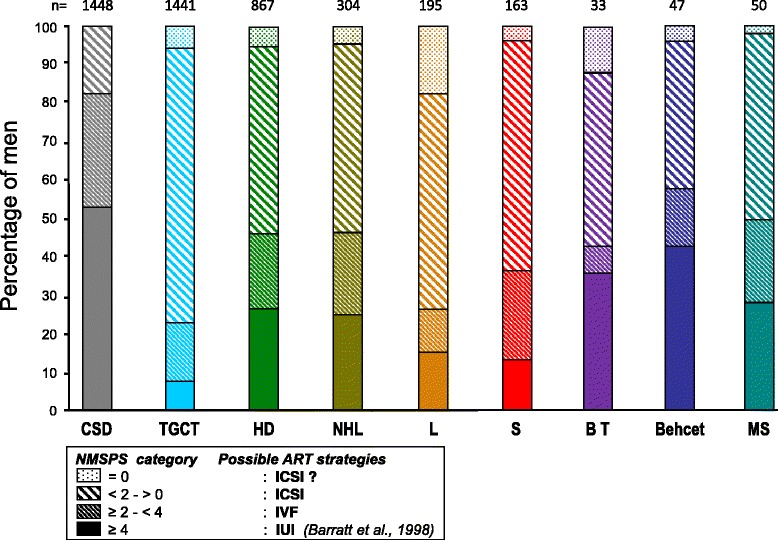
Possible ART strategies according to the pathologies studied and the number of progressive motile sperm per straw (NMSPS; ×10^6^). It is assumed that there are a sufficient number of stored straws and the fertility check-up in the female partner is normal

What were the theoretical possibilities to use banked sperm through current ART? Figure 4 summarizes the percentage of men who may benefit from IUI, IVF or ICSI according to the quantity and quality of spermatozoa within straws when grossly estimated by the number of progressively motile sperm per straw (NMSPS). Overall, an ICSI would be the required ART approach for more than 50 % of all patients, with the lowest need in Behcet patients (42 %) and the highest in TGCT patients (85 %), the theoretical use in case of CSD being 18 %. In contrast, simple IUI may be proposed in only 10 to 40 % of cases depending on the pathological condition versus 54 % in CSD patients.

## Discussion

To our knowledge, this study presents the largest series of patients referred for sperm cryopreservation before potentially gonadotoxic treatment, thus providing sound data on semen quality for various pathological conditions. The extent of poor semen quality is underlined thanks to the calculation of the percentage of normozoospermia as well as the comparison with the level of semen quality in two large groups of healthy fertile men. In addition, our study provides baseline data in cases of brain tumour, Behcet’s disease and multiple sclerosis, three pathological conditions for which possible impact on semen quality has rarely been studied.

The possible direct or indirect role of the pathologies studied on the level of semen quality is briefly discussed below. Then, we discuss the feasibility of sperm banking in the various pathologies as well as its theoretical use for a parental project when a long lasting post-treatment azoospermia is observed.

### Semen quality

Although sperm alterations most commonly occur as a result of treatment with gonadotoxic agents, they could also be observed before treatment, and may depend on cancer type. Moreover, only few studies have compared semen quality in different pathologies and healthy volunteers [19–21].

Various pathophysiological hypotheses could be raised to explain spermatogenesis impairment in a context of cancer. Those include direct tumour effects on testis and male reproductive tract, but also indirect impact with endocrine disturbances, or nutritional, autoimmune and systemic effects of cancer (see [22] for review). Sperm chromatin assay (SCSA) [23, 24] revealed sperm DNA damage due to pathology when compared to healthy fertile men. Moreover, in our study, a decrease in seminal volume was also observed for cancer or immunological disease patients, whereas the abstinence delay tends towards being longer than in fertile groups. Because semen volume depends on the contraction of the accessory glands which is influenced by the level of excitement during semen collection, these patients not being in an optimal psychological condition, this may certainly explain most of the observed low semen volumes which simply reflect a lower level of excitement. However, other additional factors such as an hypoandrogenisation for some of the patients cannot be ruled out.

Some differences can be noted according to the type of pathology. For testicular cancer, the majority of authors suggested altered semen parameters, usually sperm concentration and count [3, 4, 21, 25–28], the results being more controversial for motility and morphology. In our series, we observed significantly decreased sperm concentration, count, motility and morphology when compared to semen parameters of both groups of fertile healthy men. Only 50.9 % of men with testicular cancer presented normozoospermia when WHO 2010 reference thresholds were applied. Williams et al. found that 37 % of men with testicular cancer were normozoospermic using previous WHO 1999 criteria [21] and Hotaling et al. reported that only 59 % of TGCT patients had a total motile sperm count of more than five million [20]. However, both of these studies used low sample sizes and other reference values, making results difficult to compare. In addition to common mechanisms in all types of cancer, pre-existing defects in germ cells as part of testicular dysgenesis syndrome could also be involved in case of testicular cancer [29], as suggested by frequent histological modifications that are found in the controlateral testis [30].

For haematological diseases, most of studies revealed impaired sperm parameters. However, results are very difficult to compare because of the wide variety of diseases, leading either to small sample sizes in the studies [20, 21, 27, 28] or to a combination of different pathologies [3, 4, 19]. In our series, we observed moderate but significant decreased sperm concentration, count, motility and morphology in patients presenting lymphomas; 65.1 and 59.5 % of men had normozoospermia in cases of Hodgkin’s lymphoma or non Hodgkin’s lymphoma, respectively. On the contrary, a drastic decrease of motility and morphology were observed for men presenting leukemia, and only 36.9 % presented normozoospermia. Altered general state, hyperthermia (frequently observed during lymphomas) or testicular infiltration (as in acute leukemia), could be additional pathophysiological mechanisms responsible for the impaired spermatogenesis during haematological diseases [6, 31].

Semen quality may be expected to be better in case of chronic forms of leukemia than in case of acute leukemia due to the possible severe deterioration of the general health conditions and the impact of high fever episodes in this form of leukemia. Because we had no mean to a posteriori separate both forms of leukemia we could not provide baseline data for each subcategory. For other solid malignant diseases, literature is scarce, usually showing normal or subnormal sperm parameters [20, 21, 26, 27], but with very small sample sizes (some with a maximum of ten). For men presenting sarcoma, a moderate decrease in all semen parameters was observed in our series. On the contrary, for men presenting cerebral tumours, sperm concentration and counts were comparable to those of healthy fertile men, whereas motility and morphology were drastically reduced.

Finally, little is known about semen parameters of men presenting severe systemic diseases such as Behcet’s disease or multiple sclerosis. Altered semen parameters were pointed out in the only available study comparing 68 men with multiple sclerosis to 48 healthy volunteers [32]. For Behcet’s disease, impaired sperm production was suggested [33]. In our series, we observed moderate alterations in semen parameters for both groups. About 70 % of men presented normozoospermia according to WHO 2010 reference values.

### Clinical considerations

There is a number of barriers to sperm banking. One can ask what is the proportion of men actually referred to a sperm bank (who probably do not represent the majority of men of reproductive age likely to be exposed to gonadotoxic treatment) who could effectively benefit from this procedure?

In our series, failure to collect a sperm sample was observed in 5.7 % of men, slightly higher than in other studies with about a 3 % collection failure rate [3, 4]. This difference may be explained by the absence in previous publications of populations more exposed to collection failure, such as men with brain tumours, sarcoma or multiple sclerosis. For these pathological conditions, semen collection failure could be related to stress, but also to severe illness with very impaired general health (especially in leukemia or brain tumour populations), sexual inexperience (especially for very young adults, as in a sarcoma population), or neurological alterations (as seen in men with multiple sclerosis). Azoospermia was observed in 5.7 % of all men, being different according to different pathologies and ranging from 0 % in brain tumours to 13.2 % in leukemia. In leukemia cases, literature showed conflicting results, with azoospermia ranging from 0.8 [4] to 24 % [27], including 12.5 % [28]. Those differences may be due to small sample sizes, making results difficult to compare. In testicular cancer, we observed a rate of azoospermia of 5.0 %, mostly comparable to previously published results in lower sample sizes [3, 4, 19].

The question of the difference in the tolerance of the spermatozoa to the processes of freezing in liquid nitrogen vapours and subsequent thawing to ambient temperature in patients with various cancers compared to other groups of men has rarely been studied [34]. In our study we observed a reduced progressive motility recovery in all groups, except brain tumours and immunological diseases, when compared to fertile candidates for sperm donation. Our results concur with Caponecchia et al. who found that the percentage of surviving sperm cells was significantly lower in oncologic groups of men, especially in a group of men having leukemia, than in a fertile group (32.1 and 50.1 %, respectively) [19]. On the contrary, Agarwal et al. suggested that there was no additional loss of semen quality after thawing beyond that to be expected from any semen cryopreservation [35]. This overall decrease in the motility recovery rate contributes to the constitution of straws with significantly lower NMSPS for men presenting testicular cancer, haematological disease, sarcoma or Behcet’s disease, when compared to fertile sperm donors. Beyond discussion about minimal NMSPS required for ART [36, 37], when evaluating the theoretical use of those straws, decreased NMSPS in cancer patients leads to a less frequent use of IUI and a more frequent use of IVF with or without ICSI, regardless of the female fertility check-up or the number of available straws. For a small number of cases, no motile sperm cells were observed post-thawing, making the use of the stored semen samples uncertain, even for an ICSI attempt. In those situations, the hypo-osmotic swelling test (HOS-test) on a frozen and thawed straw constitutes a very useful adjunct to ascertain if an ICSI attempt may be programmed [38].

## Conclusions

Our study, distinguished by its high semen sample size, provides strong evidence that most of the pathological conditions we examined seriously affect sperm production and quality. However, due to its descriptive nature, it does not offer explanations on the causal links or modes of action, so more research in the field remains needed. Despite the impact of the diseases considered in the study on semen quality at the time when the patients were referred to the sperm bank, spermatozoa may be cryopreserved for the vast majority of referred men. This provides opportunity for future paternity in cases of post-treatment definitive azoospermia due to different ART techniques, especially ICSI, which has significant success rates even when initial levels of sperm production and quality or sperm survival rates are low. While post-treatment recovery in sperm production is often found, depending on type of treatment, age and individual factors, sperm cryopreservation before any possibly gonadotoxic therapy remains the gold standard for fertility preservation. This preventive care should be proposed to all patients concerned.

## Abbreviations

ART: assisted reproductive technologies
BT: brain tumour
CECOS: Centre d’Etude et de Conservation des Œufs et du Sperme humains (the public French federated sperm, oocyte and embryo banks)
CSD: candidate for sperm donation
HD: Hodgkin disease
HOS-test: hypo-osmotic swelling test
ICSI: intra cytoplasmic sperm injection
IQ: inter quartile (range)
IUI: intra-uterine insemination
IVF: in vitro fertilization
L: Leukemia
MS: multiple sclerosis
NHL: non Hodgkin lymphoma
NMSPS: number of motile sperm per straw
PPW: partner of pregnant women
S: sarcoma
SCSA: sperm chromatin structure assay
TGCT: testicular germ cell tumour
WHO: World Health Organization
